# Activation of Nrf2 to Optimise Immune Responses to Intracerebral Haemorrhage

**DOI:** 10.3390/biom12101438

**Published:** 2022-10-07

**Authors:** James J. M. Loan, Rustam Al-Shahi Salman, Barry W. McColl, Giles E. Hardingham

**Affiliations:** 1 Centre for Discovery Brain Sciences, University of Edinburgh, Edinburgh EH8 9XD, UK; 2 UK Dementia Research Institute at Edinburgh, University of Edinburgh, Edinburgh EH16 4SB, UK; 3 Centre for Clinical Brain Sciences, University of Edinburgh, Edinburgh EH16 4SB, UK

**Keywords:** astrocytes, bardoxolone methyl, dimethyl fumarate, inflammation, intracerebral haemorrhage, Nrf2, macrophages, microglia, monocytes, omaveloxolone, oxidative stress, perihaematomal oedema, sulforaphane, transcription factor

## Abstract

Haemorrhage into the brain parenchyma can be devastating. This manifests as spontaneous intracerebral haemorrhage (ICH) after head trauma, and in the context of vascular dementia. Randomised controlled trials have not reliably shown that haemostatic treatments aimed at limiting ICH haematoma expansion and surgical approaches to reducing haematoma volume are effective. Consequently, treatments to modulate the pathophysiological responses to ICH, which may cause secondary brain injury, are appealing. Following ICH, microglia and monocyte derived cells are recruited to the peri-haematomal environment where they phagocytose haematoma breakdown products and secrete inflammatory cytokines, which may trigger both protective and harmful responses. The transcription factor Nrf2, is activated by oxidative stress, is highly expressed by central nervous system microglia and macroglia. When active, Nrf2 induces a transcriptional programme characterised by increased expression of antioxidant, haem and heavy metal detoxification and proteostasis genes, as well as suppression of proinflammatory factors. Therefore, Nrf2 activation may facilitate adaptive-protective immune cell responses to ICH by boosting resistance to oxidative stress and heavy metal toxicity, whilst limiting harmful inflammatory signalling, which can contribute to further blood brain barrier dysfunction and cerebral oedema. In this review, we consider the responses of immune cells to ICH and how these might be modulated by Nrf2 activation. Finally, we propose potential therapeutic strategies to harness Nrf2 to improve the outcomes of patients with ICH.

## 1. Introduction

Bleeding into the brain can be catastrophic. In adults, this commonly occurs because of spontaneous rupture of a parenchymal arteriole, resulting in spontaneous intracerebral haemorrhage (ICH) [1]. This is the most common cause of haemorrhagic stroke, which can also be due to spontaneous bleeding into the subarachnoid space, termed subarachnoid haemorrhage (SAH), usually due to rupture of an intracranial aneurysm [2]. ICH may also arise as a consequence of rupture of an arterial aneurysm or other vascular malformation, trauma or surgical injury [3,4,5,6]. Together, this accounts for a huge burden of death and disability; stroke is estimated to be the second leading cause of death worldwide and the third largest cause of death and disability combined, of which ICH contributes a disproportionately high burden of death and disability [5,7]. Although age-standardised global ICH incidence has declined since 1990, this is driven by high-income countries, which masks static or rising incidence rates in low-middle income populations, which are disproportionally burdened by ICH as a proportion of all stroke [5,8]. 

## 2. Intracerebral Haemorrhage

To establish the pathophysiological consequences of ICH, it is useful to consider a relatively common and homogenous disease entity. Therefore, in this review we will focus on spontaneous ICH. This arises from bleeding from a ruptured diseased arteriole (e.g., affected by hypertensive arteriosclerosis or cerebral amyloid angiopathy [1]). The major risk factor for ICH is systemic arterial hypertension [5,8]. Increasing age and male sex are associated with higher blood pressure and therefore are also associated with ICH [8,9,10,11]. Measures to reduce haematoma volume by reducing bleeding using haemostatic agents or rapid blood pressure lowering and by surgical evacuation of haematoma have not been shown to be effective in improving primary outcome measures in high-quality randomized controlled trials (RCTs) [12,13,14,15,16,17]. There are currently no effective medical or surgical interventions for ICH. 

Overall outcome after ICH is poor. Case fatality at one month is approximately 40%, 54% at one year, 71% at five years and 82% at 9 years [18,19,20,21]. At three months approximately 26% of all incident patients are functionally independent, falling to 15% at one year [21,22,23]. The association of haematoma volume with long term survival and outcome largely is mediated through its association with early mortality and disability, with subsequent decline possibly being attributable to processes of secondary brain injury as well as the risk of major adverse cardiovascular events [21,24,25]. Whilst peak perihaematomal oedema, which occurs around two weeks from ICH onset, has been associated with poor long-term functional outcome, following adjustment for haematoma volume, early oedema volume at three days or earlier is not [21,26]. This aligns with a longitudinal study of transcriptional responses of haematoma derived immune cells which identified a window of intense transcriptional activity up to four days after ICH onset, which was associated with functional outcome [27]. There may therefore be an early 3–4 day window after ICH onset during which the responses of immune cells could be modulated to minimize, or protect against, secondary injury.

## 3. Secondary Injury

There are limited conclusive data to inform our knowledge of the processes of secondary injury after ICH in humans [28,29]. Consequently, our collective understanding is largely derived from animal studies or single studies of human brain tissue. 

### 3.1. Haemorrhage Propagation

Early haematoma expansion occurs due to ongoing bleeding from the initial rupture site, as well as from rupture of other blood vessels disrupted by the primary haemorrhage. This is maximal within the first three hours from ICH onset [30]. Haematoma expansion causes tissue division as well as compression and kinking of other blood vessels plus release of endogenous collagenases which degrade the basal lamina, compromising the blood brain barrier [31]. 

### 3.2. Cytotoxicity and Oxidative Injury

In rodents, cytotoxic injury in the brain occurs due to the accumulation and lysis of haem-laden erythrocytes, monocytes, neutrophils and platelets [27,32,33,34]. In particular, ferrous iron (Fe^2+^), a component of haem which is released from lysed erythrocytes, is potently cytotoxic [35]. Haem-associated cytotoxicity occurs through ferroptosis, iron-dependent programmed cell death, whereby haem-Fe interacts with hydrogen peroxide to drive the production of free radicals in a Fenton-like reaction, which causes lipid peroxidation and glutathione depletion [35,36,37]. In preclinical models, and patients with subarachnoid haemorrhage, free extracellular oxyferrous haemoglobin exacerbates secondary ischaemia by oxidising nitric oxide (NO) to nitrate (NO_3_^−^) [38,39,40]. NO is a potent vasodilator, and its loss is associated with a propensity for spreading cortical depolarisation and ischaemia [41]. Hypoxia in turn increases oxidative stress by altering mitochondrial metabolism to favour reactive oxygen species (ROS) generation [42]. This mechanism has been localized to mitochondrial complex III, with hypoxia possibly driving an increase in the lifetime of ubisemiquinone, thus favouring electron donation to oxygen, resulting in intracellular superoxide generation [43]. Further, release of intracellular glutamate by erythrocytes and other cells may increase the extracellular glutamate concentration significantly, driving excitotoxic neuronal injury [44,45].

## 4. Immune Responses

Immune responses to ICH are heterogenous with the potential to exert harmful as well as helpful actions on brain tissue viability and function. This provides an opportunity to develop therapeutics which maximise protective responses, whilst minimising harmful ones, by targeting upstream molecular regulators of responses to ICH that direct neuroprotective and neurorestorative pathways. 

### 4.1. Myelomononuclear Cells

Haematoma formation, as well as the associated cell injury and death initiate a marked immune response. Microglia are a self-renewing population of central nervous system resident parenchymal macrophages which are derived from the yolk sac and comprise the major immune cell type of the uninjured brain parenchyma [46,47,48]. In rodent models of ICH, these are joined by cells recruited from the circulating peripheral blood monocyte population and, likely with meningeal, choroid plexus and perivascular macrophages [49,50], these myelomononuclear cells execute the initial innate immune response to ICH [49].

In rodent models of ICH, microglia are stimulated by the activation of molecular pattern recognition receptors, including toll-like receptor 4 (Tlr4), which recognises haematoma constituents including haem and fibrinogen [51,52,53]. Further, death and injury of brain cells and erythrocyte lysis results in the release of so-called damage-associated molecular patterns (DAMPs), and intracellular cytokines [54,55,56]. These factors stimulate microglia which, in patients with ICH, rapidly transition from a surveillant ramified appearance to a reactive morphology with shortened processes and enlarged cell bodies [57]. In rodents, reactive microglia both proliferate and migrate to the injured region where they phagocytose cell debris and secrete chemokine ligand 2, a potent chemoattractant, and hydrogen peroxide, a ROS that induces microglial proliferation [58,59,60]. 

Studies of both rodents and humans have shown that monocyte derived cells (MdCs) are also recruited to the haematoma region. These may be directly derived from the haematoma, having been sequestered there during the initial haemorrhage, or actively recruited from the peripheral blood circulation or central nervous system “border” regions [27,49,50,61]. In rodent models of peritonitis and wounding, recruited monocytes recruited begin to differentiate into tissue macrophages or dendritic cells within 18h [62,63]. Studies of the evolving transcriptional profile of peripherally derived brain and haematoma monocytes suggest that a similar process occurs in rodents and patients with ICH, respectively, although no definitive lineage tracing study has yet been performed [27,33,49,61].

Initially, perihaematomal microglia and MdCs in rodents express high levels of transcripts for proinflammatory cytokines *Tnf, Il1a* and *Il1b* [33,49,59]. In patients, high levels of IL1B are also detectible from 6h after injury, potentially representative of this initial myelomononuclear response [28]. However, although in mice depletion of “classically reactive” Ccr2+ monocytes is initially associated with reduced neurological deficits and neuronal injury, at later time points Ccr2^+^ MdCs contribute to haematoma resolution and functional recovery by Axl-dependent efferocytosis of eryptotic erythrocytes [33,49,60].

In patients and mice, microglia and MdCs contribute to haematoma resolution by phagocytosis of haematoma components and dead cells [33,57,64]. In doing so, the lysosomal components of these cells become expanded, resulting in a lipid-laden “foamy” appearance [57]. Uptake of extracellular haptoglobin-haemoglobin complexes by myelomononuclear Cd163 and lysosomal breakdown of haematoma drives a rise in intracellular iron concentration [39]. In animal models, this transition to a phagocytic state is associated with a reduction in the expression of proinflammatory cytokines and increased expression of protective factors, including haem oxygenase 1, a critical factor for haem detoxification [33,52,65]. 

### 4.2. Neutrophils

Neutrophils are polymorphonuclear granulocytes that infiltrate the brain within the first two days after ICH in both humans and rodent ICH models, but are less numerous than myelomononuclear cells at all time points [57,66,67]. Nonetheless, these cells mediate distinct immune responses to ICH and have both secretory and phagocytic properties [68]. Neutrophils can generate high concentrations of ROS in a process termed “oxidative burst”. This serves to degrade extracellular debris and also augments the production of other factors secreted by neutrophils, including proinflammatory cytokines and neutrophil extracellular traps (NETs) during a process termed NETosis [69,70]. NETs are organised webs of decondensed chromatin that are released by activated neutrophils largely in a process of organised cell death (termed NETosis) and exhibit proinflammatory, haemostatic and bactericidal properties [71,72,73,74].

Neutrophil degranulation and release of secretory vesicles results in the extracellular accumulation of proinflammatory cytokines, metalloproteinases, and iron and haemoglobin binding molecules [70,75]. Because neutrophils secrete chemoattractants, which augment early monocyte recruitment, and ROS, which are histotoxic, neutrophil depletion is associated with improved early functional outcome in mice with ICH [76]. As described previously regarding “classical monocytes”, it is possible that although early neutrophil depletion is protective at early stages, this might have harmful later consequences [33]. 

### 4.3. Lymphocytes

Broadly, lymphocytes can be categorised as B-cells, T-cells or natural killer (NK) cells. These cell types orchestrate adaptive immune responses [77]. NK cells are lymphocyte effectors of innate immunity, that respond to tumours and viral infections by triggering death of affected cells and release of cytokines [78]. 

Little is known about the effects of ICH on lymphocytes. In one study, CD3^+^ T-cells were present in the haematomas of patients with ICH, but were few in the parenchyma up to 12 days after ICH [57]. In contrast, another smaller qualitative study of patient brain tissue found increased CD3^+^ cells in perihaematomal tissue, which were in proximity to blood vessels and dendritic cells [79]. In studies of rodent ICH models, both Cd4^+^ helper and Cd8^+^ cytotoxic parenchymal effector T-cells have been reported to be increased early after ICH [80,81]. A deeper literature exists for ischaemic stroke, where effector T-cell migration to ischaemic brain tissue is driven by recognition of a brain autoantigen released across the disrupted blood-brain barrier [82,83]. In this context, T helper-1 cells might contribute to blood brain barrier dysfunction and oedema by secretion of proinflammatory cytokines [84]. Conversely peripheral infusions of regulatory T-cells, which induce tolerance of autoantigens, in mice with ICH resulted in improved neurological deficit and reduced brain cytokine and matrix metalloproteinase expression [85]. These findings indicate that modulation of T-cell subclasses after ICH could be a therapeutic avenue. However, as it is not currently established if, and how, T-cells respond to ICH in humans, the translational application of findings from rodent studies is limited. 

Whilst our collective understanding of any roles of T-cells in the brains of patients or animals with ICH is limited, knowledge of B-cell and NK cell responses is even more so. One analysis of mice with ICH found small numbers of B220^+^ B-cells in their brain tissue, but this did not have an association with time from ICH onset [86]. It is unclear if B-cells or NK cells are recruited to perihaematomal brain tissue in humans. 

### 4.4. Astrocyte Responses

Astrocytes are one of the most abundant glial cell types of the central nervous system [87]. Astrocytes derive from radial glia, exhibiting a fully mature state of differentiation by 6–12 months postnatally in humans [88]. They perform a wide range of actions that are instrumental to the function of the central nervous system in health and disease [89,90]. They provide homeostatic support for neurons by glutamate reuptake, lactate, cholesterol and glutathione precursor production as well as glycogen storage and regulation of perivascular water transport, ionic and pH homeostasis, clearance of amyloid β and neurovascular coupling [91,92,93,94,95,96,97,98,99]. Moreover astrocytes have important functions in acute brain injury which integrate with those of immune cells. 

In patients with ICH, GFAP^+^ reactive astrocytes accumulate in perihaematomal regions and are colocalised with NF-𝜅B-positivity [100,101,102,103,104]. In mice, reactive astrocytes similarly surround the haematoma which is associated with their expression of matrix metalloproteinases [105]. There is a lack of detailed analyses of specific functional or secretory responses of astrocytes to ICH. However, studies of astrocytes in other models of trauma, hypoxia, oxidative stress and inflammation demonstrate that astrocytes respond to a wide variety of stressors. Astrocytes sense injury and inflammation through their expression of pattern recognition receptors, cytokine receptors as well as intracellular sensors of hypoxia and oxidative stress [106,107,108,109]. On activation, astrocytes adopt myriad reactive phenotypes [110,111,112]. They can secrete long-chain free fatty acids which are toxic to neurons and oligodendrocytes in vitro and impair resilience to axonal injury in vivo [113]. They are capable of expressing proinflammatory cytokines, vasoactive peptides, complement as well as interferon and thus contribute to the cerebral oedema and the inflammatory milieu [114,115,116,117]. In mice with traumatic brain injury, disruption of perivascular fluid and solute clearance from the brain may also contribute to oedema formation and accumulation of amyloid [95]. In addition to being directly influenced by tissue damage, the balance between neuroprotective and harmful astrocyte phenotypes may be modulated by the expression of cytokines by monocytes [113,118]. Additionally, other critical homeostatic functions of astrocytes, including glutamate reuptake, neurite phagocytosis, ion buffering and neuron-astrocyte metabolic coupling are lost or impaired following a range of insults [110,119].

Astrocyte responses also be adaptive. Following acute brain injury, astrocytes migrate to the site of injury and are major contributors to glial scar formation. Although this has historically been viewed as a barrier to neuroregeneration, particularly in spinal cord injury, there is accumulating evidence to suggest that, in mice, such astrogliosis may support axonal regrowth by the expression of supportive extracellular matrix and cell adhesion proteins [120,121]. Astrocytes, which in steady state undertake a degree of synaptic pruning by phagocytosis, may acquire a brief period of enhanced phagocytic capacity to engulf cell debris after experimental ischaemic stroke [122]. 

These studies of rodent models of conditions other than ICH astrocytes respond to relevant harmful stimuli in ways that both harmful and protective. It is not currently clear whether these processes occur in rodents or patients with ICH. This is a priority for further study.

### 4.5. Summary

**Figure 1:**
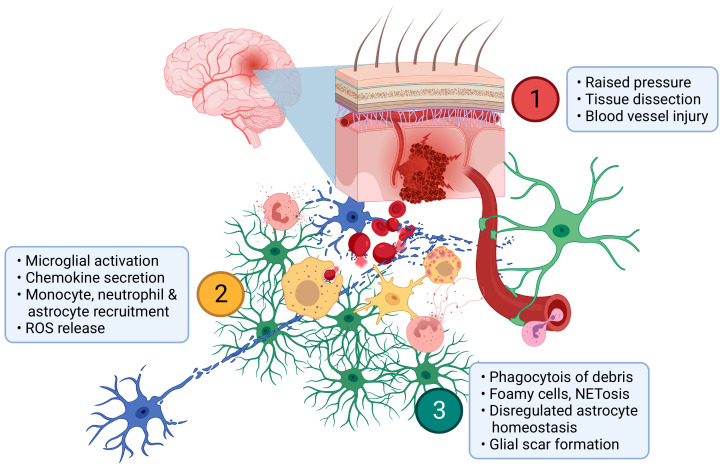
**Pathophysiology of ICH.** (**1**) Haemorrhage from a parenchymal arteriole drives pressure gradients that dissect neural tissue (blue) and may cause distant injury as well as blood vessel injury. (**2**) Myelomononuclear cells (yellow) rapidly respond by release of proinflammatory cytokines and other chemokines. These serve to recruit MdCs, astrocytes (green) and neutrophils (amber) which secrete further inflammatory mediators and reactive oxygen species (ROS). (**3**) As inflammation progresses, debris is progressively phagocytosed and foamy myelomononuclear cells appear. Neutrophil NETosis may serve to limit haemorrhage and/or microvascular blood flow. As astrocytes respond to the ICH and contribute to glial scar formation, their homeostatic functions including neurovascular and neurometabolic coupling are impaired. Created with BioRender.com.

ICH causes a mechanical primary injury as the evolving haematoma dissects tissue and exerts pressure on distant structures (Figure 1). This is rapidly followed by cytotoxicity due to haem release from erythrocytes and ischaemia. The innate immune response is initiated by microglia which respond to cell injury and death as well as the highly oxidative environment by secreting chemotactic agents, phagocytosing debris and inducing cytoprotective factors. Neutrophils, monocytes/MdCs and astrocytes are then recruited to the perihaematomal region within days of ICH onset and contribute to the secretion of inflammatory factors, phagocytosis of debris and metabolic support of neurons [27,33,49]. Over time, proinflammatory actions of myelomononuclear cells and astrocytes wane, neutrophil numbers decline and a glial scar forms. 

## 5. NF-E2-Related Factor 2 (Nrf2)

Nrf2 is a basic leucine zipper transcription factor belonging to the Cap ‘N’ Collar family that is encoded by the *Nfe2l2* gene [123]. Nrf2 directly and indirectly influences the expression of thousands of genes encoding antioxidant, cytoprotective and proinflammatory proteins and, as such, is a potential therapeutic target for ICH [39,124,125].

### 5.1. Structure, Regulation, and Expression

*NFE2L2* is expressed by almost all human tissues and cell types [88,126]. In the uninjured adult human brain, it is most highly expressed by myelomononuclear cells but, notably, is epigenetically repressed in neurons and what little is made is rapidly degraded [88,127,128,129,130,131].

**Figure 2:**
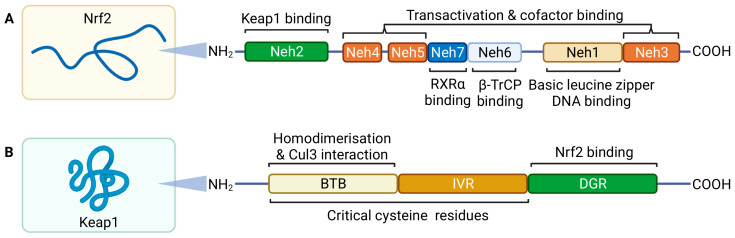
**Nrf2 and Keap1 structure.** Nrf2 (**A**) is comprised of seven functional Neh domains. Neh2 is required for Keap1 dependent homeostatic regulation. Neh3, Neh4, and Neh5 are required for transactivation and facilitatory cofactor binding and Neh1 contains the DNA binding basic leucine zipper domain. Neh6 and Neh7 contain sites that allow redox independent Nrf2 inhibition. Keap1 (**B**) contains two major domains that are relevant to Nrf2-regulation. The broad complex, tramtrack and bric a brac (BTB) domain is required for formation of the Keap1 homodimer-Cul3 complex and the double glycine repeat (DGR) anchors Nrf2 and actin to maintain Nrf2s cytoplasmic localisation during steady state. Oxidation of cysteine residues located in the BTB and intervening region (IVR) result in conformational change that releases Nrf2. Adapted from Jaramillo and Zhang (2013) [132]. Created with BioRender.com.

Nrf2 protein is comprised of seven functional domains, termed Nrf2-ECH homology (Neh) 1-7 (Figure 2) [132]. The first N-terminal domain is Neh2, which contains regions that regulate the stability and activity of Nrf2 [132]. Seven lysine residues are contained within Neh2 and these serve as sites for ubiquitination by the Cullin 3 (Cul3)-dependent E3 ubiquitin ligase complex RING box protein 1 (Rbx1) [133]. This leads to proteasomal degradation of Nrf2 and thus maintains cytoplasmic Nrf2 levels at steady state in constitutive conditions and prevents its nuclear localisation [133,134]. Ubiquitination of Nrf2 at Neh2 by the Cul3-Rbx1 complex is regulated by the homodimeric adaptor protein Kelch-like ECH-associated protein 1 (Keap1) [132,133,135]. Keap1 anchors Nrf2 to cytoplasmic actin through Keap1’s Kelch domain, also known as the double glycine repeat (DGR) domain [136]. Two binding sites in the Neh6 domain of Nrf2 allow for binding of β-transducin repeat containing protein (β-TrCP), an adaptor for an alternative ubiquitin ligase complex, Skp1-Cul1-Rbx1 [137]. This potentiates phosphorylation of Nrf2 by glycogen synthase kinase-3β [137,138]. Additionally, the retinoic X receptor ⍺ (RXR⍺) can interact with the Neh7 domain, competing with chromatin binding to inhibit transactivation by Nrf2 [139]. These provide redox-independent modes of Nrf2-regulation. However, the physiological significance and role of these are unknown.

**Figure 3:**
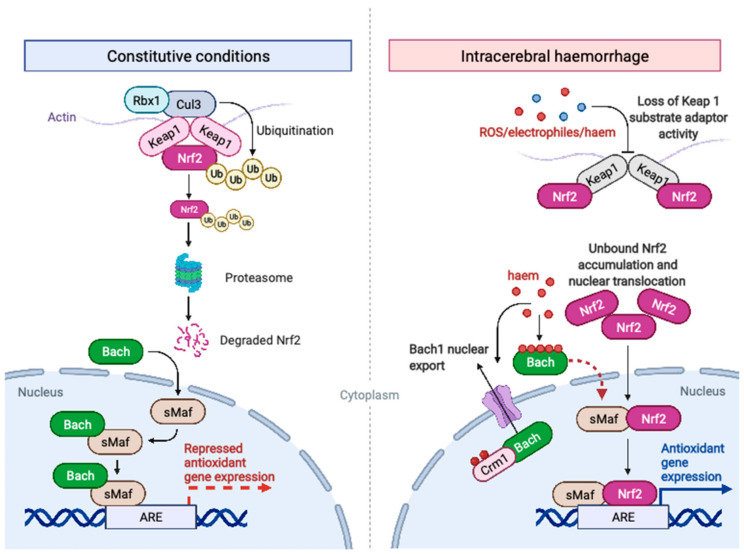
**Regulation of Nrf2 activation in constitutive conditions and after ICH.** Under constitutive conditions, Nrf2 is anchored to cytoplasmic actin by Kelch-like ECH-associated protein 1 (Keap1) homodimers. These also function as adaptor proteins for Cullin 3 (Cul3)-dependent E3 ubiquitin ligase complex RING box protein 1 (Rbx1) which ubiquitinates Nrf2, leading to its proteasomal degradation. BTB and CNC homology (Bach) proteins bind nuclear small Maf (sMaf) protein and repress expression of certain antioxidant genes. During conditions of oxidative stress or haem accumulation, as may occur after ICH, Keap1 loses its’ substrate adaptor activity and becomes saturated with Nrf2. Nrf2 levels rise and Nrf2 localises to the nucleus where it heterodimerises with sMaf proteins to transactivate genes containing an antioxidant response element (ARE) motif. Simultaneously, haem binding of Bach proteins prevents heterodimer formation with sMaf proteins and induces nuclear export of Bach by chromosomal maintenance 1 (Crm1). Created with BioRender.com.

Under conditions of oxidative stress, which may be induced by ROS, haem or electrophilic stimuli, including heavy metal ions such as iron released from haem, Nrf2 accumulates intracellularly. This is because of loss of the substrate adaptor activity of Keap1 resulting from oxidation of cysteine residues of the broad complex, tramtrack and bric a brac (BTB) and the intervening regions (IVR) of Keap1, as well as of cysteine residues on Nrf2 itself [140,141,142]. Available Keap1 consequently becomes saturated with Nrf2 protein (Figure 3) [134,135]. With available Keap1 saturated, Rbx1 fails to ubiquitinate Nrf2, intracellular Nrf2 levels rise and Nrf2 is imported to the nucleus [133,143]. In the nucleus Nrf2 competes with BTB and CNC homology (Bach1 and Bach2) protein to form heterodimers with the small musculoaponeurotic fibrosarcoma (sMaf) proteins MafF, MafG and MafK [144,145]. Heterodimerisation of Nrf2 with sMaf proteins allows efficient binding of Nrf2 with DNA at regions containing an antioxidant response element (ARE) motif [145,146]. In addition to competitive repression of Nrf2 activity, heterodimerised Bach proteins, of which Bach1 is dominant in human myelomononuclear cells, directly binds and represses transcription of a subset of genes sharing the ARE motif [88,147,148]. Bach protein thus serves as a dominant negative regulator of Nrf2 activity [149]. However, an increase in intracellular haem concentration, as occurs after ICH, serves to directly oppose the transrepression activity of Bach. Haem binds directly to Bach proteins, interfering with the formation of Bach-sMaf heterodimers [150,151]. Moreover, haem interacts with a nuclear export signal present on Bach1 and Bach2, resulting in their export from the nucleus by the nuclear exporter Crm1 [152]. Additionally, oxidative stress increases the transactivation potential of the Nrf2 Neh5 domain, which is another potential Keap1-independent mode of Nrf2 activation after ICH [153]. The net effect of these processes is that Nrf2 is activated in tissue close to the haematoma surface in patients with ICH [153]. Notably Nrf2 localisation after ICH is submaximal [153]. This may reflect preclinical findings that the transcriptional response to Nrf2 activation wains with age and that proinflammatory responses to ischaemic stroke become more marked [154,155]. 

### 5.2. Transcriptional Regulation by Nrf2

Nrf2 influences the transcription of thousands of genes in fashions that are stimulus-, cell type-, and tissue-dependent, involving mechanisms requiring direct chromatin binding and through indirect influences on cellular state and other transcription factors [27,88,156,157].

Nrf2-sMaf heterodimers directly induce the transcription of genes with ARE motif-containing promoter regions [146]. This drives a relatively stereotyped, evolutionally conserved, core programme of gene expression [158,159]. Genes directly induced by Nrf2 include enzymes that catalyse catabolic and anabolic reactions, as well as those which support redox buffering and metabolism, as well as the reversal of oxidative damage (Table 1) [160,161,162]. Notably, Nrf2 directly induces the expression of several factors required for the sequestration, uptake and detoxification of haemoglobin and iron which may be protective after ICH. These include, but are not limited to, haem oxygenase 1, biliverdin reductase B, ferritin and haem transporter HRG1 [39,124,163,164].

In addition to direct transactivation, Nrf2 exhibits some direct transrepression activity by binding promotor regions of certain proinflammatory factors and preventing recruitment of RNA polymerase II [125]. Further, Nrf2 indirectly affects the expression of a wider repertoire of genes. After induction of electrophilic stress by administration of a xenobiotic, Nrf2 dependent transrepression is not detectible until 24 h, whilst transactivation is detectible at 6 h [165]. This temporal separation of responses to electrophilic stress suggests that, in this context, transrepression is indirectly mediated by protective effect of ARE gene induction. By competing for sMaf proteins, Nrf2 may also inhibit activation of the NF-𝜅B subunit p65 [166,167,168]. 

This demonstrates that Nrf2 both facilitates a cytoprotective antioxidant response and suppresses, through context-dependent direct and indirect means, the expression of certain inflammatory mediators. Therefore, activation of Nrf2 may optimise adaptive cellular responses to ICH, by augmenting protective elements whilst suppressing responses that might contribute to secondary injury. 

## 6. Therapeutic Modulation of Nrf2

Augmentation of Nrf2-mediated responses to haemorrhagic brain injury is a potential therapeutic strategy, particularly because older populations with ICH may not exhibit a maximal Nrf2-mediated transcriptional response. Various Nrf2-activating drugs exist and their therapeutic efficacy and safety has been examined in studies of both rodents with ICH and patients with other disease states. Nrf2-activating drugs trialled in patients and animal models of ICH all function through a common mechanism involving electrophilic modification of cysteine residues on Keap1 [169].

### 6.1. Preclinical Studies

Nrf2 has been considered by several studies of rodent and in vitro models of ICH (Table 2). These have consistently demonstrated that global Nrf2 deficiency (Nrf2-/-) causes worse outcomes and that all 11 putative Nrf2 activators studied to date after ICH improve outcome [170]. However despite the compounds improving outcome, the mechanistic basis for their protection is unclear. Reviews of Nrf2 activator use in vivo to date are narrative, do not use a systematic approach to study identification and do not consider publication bias [170]. Caution is required to avoid overinterpretation of such analyses.

ICH volumes in Nrf2-/- rodents have been demonstrated to be greater than in wild type (WT) counterparts from as early as 24 h [171,172]. One study using an Nrf2 activating drug has reported a reduction in haematoma volume in the drug treated group [172]. The link between lower ICH volumes and Nrf2 activation was therefore attributed to increased erythrophagocytosis. However studies of Nrf2-/- rodents are challenging to interpret as they exhibit compromised erythrocyte integrity which may confound analysis of haematoma volumes [124,173]. Nonetheless, there is in vitro evidence that Nrf2 activation may increase the erythrophagocytic capacity of microglia and blunt hydrogen peroxide production [172]. 

Other potential protective mechanisms of Nrf2 after ICH have also been considered. Nrf2-/- mice exhibit greater neutrophil recruitment to the perihaematomal region at 24 h and similar recruitment of myelomononuclear cells [171]. This was associated with greater amounts of peroxynitrite, a highly oxidative compound formed by the interaction nitric oxide with haemoglobin [39,171,174]. Post-injury administration of the Nrf2-activating drugs dimethyl fumarate or tert-butyl hydroquinone reduced perihaematomal IBA1 staining, interleukin-1β transcription, blood-brain barrier opening, intracellular adhesion molecule-1 protein expression and brain water content whilst increasing the expression of haem scavenging molecules CD36, CD163 and haptoglobin [175,176,177]. Treatment with other less established, or less specific, Nrf2 activators in rodent models of ICH and subarachnoid haemorrhage has yielded concordant results [39,170]. Further, activation of Nrf2 by oxidative stressors of in vitro and in vivo confers resilience to future severe hypoxia [178,179].

These rodent studies provide early evidence that the activation of Nrf2 in phagocytic cells after ICH may be protective by enhancing their phagocytic capacity, ability to withstand oxidative stress, and by suppressing proinflammatory signalling. They therefore support the use of Nrf2 activators in trials of patients with ICH. Whilst analyses have focused on the influence of Nrf2 activation or deficiency on brain immune cells, it is important to note that non-specific Nrf2 activation and deficiency may exert myriad indirect effects due to the widespread expression of Nrf2 in the brain and elsewhere [88,126]. Nrf2 activators have been trialled in patients with other conditions, giving important insights into their safety, efficacy and pharmacokinetics in humans. 

### 6.2. Clinical Studies

Various Nrf2 activators have also been the subject of randomised controlled trials (RCTs) in patients with both neurological and non-neurological disease, although none has yet been conducted in patients with ICH (Table 3). 

Dimethyl Fumarate (DMF) is a synthetic Nrf2-activating drug which is recommended for use by the UK National Institute for Health and Care Excellence (NICE) to reduce relapses and improve quality of life in relapsing-remitting multiple sclerosis [180,181,182,183]. It increases Nrf2 activation by modifying cysteine residues on Keap1, and thus increasing the nuclear translocation of Nrf2 [184]. It has been shown to improve neurological function in rodents with ICH whilst increasing expression of Nrf2 target genes and suppressing interleukin-1β and inducible nitric oxide synthetase [177,185]. Although the protective effect of DMF in animal models of multiple sclerosis is established to be Nrf2-dependent, there is minimal direct evidence of this in patients [186,187]. Further, certain canonical Nrf2 target genes have been shown to be induced by DMF in Nrf2 deficient mice, indicating that Nrf2-independent mechanisms of action may also exist [188]. Nonetheless, DMF treatment is associated with reduced numbers of interferon-𝛾 producing T-helper cells and increased T-regulator cells, a phenotype which may be protective in rodent ICH models [85,187,189]. 

Sulforaphane is an isothiocyanate which can be derived from cruciferous vegetables. This activates Nrf2 through cysteine modification of Keap1 and also modulates gene expression more broadly through the action of its two major metabolites, sulforaphane-cysteine and sulforaphane-N-acetylcysteine which are histone deacetylase inhibitors [140,190,191,192]. Following extensive study in animal models of ICH and subarachnoid haemorrhage, where it was shown to reduce inflammation and optimise certain measures of inflammatory responses, sulforaphane became the subject of a phase 2 RCT in aneurysmal subarachnoid haemorrhage [39,170,193]. This has not yet reported results [193]. 

**Table 3:** Key randomised controlled trials of Nrf2 activators in patients.

Intervention	Trial	Population	Comparator	Outcome
Dimethyl Fumarate	CONFIRM phase III trial [182]	Adults aged 18–55 with relapsing-remitting multiple sclerosis	Placebo	Reduced annualised relapse rate with dimethyl fumarate with treatment
	DEFINE phase III trial [183]	Adults aged 18–55 with relapsing-remitting multiple sclerosis	Placebo	Reduced two-year relapse rate with dimethyl fumarate with treatment
Sulforaphane	SAS Phase II trial [193]	Adult aneurysmal subarachnoid haemorrhage	Placebo	No data reported.
Bardoxolone methyl	BEACON phase III trial [194]	Adult type 2 diabetes mellitus and stage 4 chronic kidney disease	Placebo	No effect of treatment on progression to end stage renal failure. Increased risk of cardiovascular events with treatment.
	BEAM phase II trial [195]	Adult type 2 diabetes mellitus and stage 3b-4 chronic kidney disease using an angiotensin receptor blocker	Placebo	Improved one-year estimated glomerular filtration rate with treatment
Omaveloxolone	MOXIe phase II trial [196]	Adults aged 16–40 with Friedreich Ataxia and no cardiac disease	Placebo	Improved 48-week modified Friedreich’s Ataxia Rating Scale score with treatment

Nrf2 activators have been used by RCTs in non-neurological diseases. Certain triterpenoids exhibit potent and specific Nrf2-activating activity, through cysteine modification on Keap1, as well as central nervous system oral bioavailability [129,197]. One such compound, the methyl ester of 2-cyano-3,12-dioxooleana-1,9(11)-dien-28-oic acid (CDDO-Me), or bardoxolone methyl, has been the subject of RCTs in chronic kidney disease [194]. However, a major phase three trial was terminated early because of an increased risk of cardiac events associated with the CDDO-Me arm [194]. This was determined retrospectively be a probable consequence an of off target effect of the drug causing endothelin antagonism causing a worsening of fluid overload, which the trial population was vulnerable to [198]. As such, subsequent trials of alternative triterpenoid compounds, which may have less of an effect on endothelin signalling, has focused on less vulnerable populations, including patients with neurological disease. 

One of these trials used a second-generation triterpenoid derivative of CDDO-Me, omaveloxolone. This family of drugs exhibit greater suppression of interferon stimulated nitric oxide production and greater Nrf2 target gene induction than their CDDO-Me counterparts as well as differing pharmacokinetics [199]. Omaveloxolone has been subject to a phase 2 RCT in Friedreich’s Ataxia, a condition driven by dysfunctional iron metabolism, leading to mitochondrial dysfunction and oxidative injury [199,200]. This study identified an improvement in the primary efficacy outcome of a standardised assessment of neurological function, as well as an acceptable safety profile [196]. Treatment with CDDO-Me derivatives might therefore be effective after ICH, by augmenting Nrf2-dependent induction of cytoprotective functions and suppressing inflammatory gene induction. Given the potential for cardiac events with use of CDDO derivatives, further evaluation of the relative risks of other triterpenoids in older populations, which are prone to ICH, cardiac and chronic kidney disease, or exclusion of patients with a history of relevant cardiac or kidney conditions is necessary [5,11,201].

It is possible that non-specific pharmacological Nrf2 activation may have an excessive side effect profile to permit use in patients with ICH. Nrf2 activators targeted to specific cell types might show increased potency at the active site with fewer off-target effects. One approach to achieving this could be to stimulate the proliferation of monocytes or microglia cell types which express high levels of Nrf2 [88,202]. One could condition circulating peripheral leukocytes using an Nrf2 activator with low brain penetrance. These conditioned cells may then enter perihaematomal brain tissue in a protective Nrf2 activated state. Alternatively, one might target drugs to mononuclear phagocytes by encapsulating them in liposomes or incorporating them with lipid-based nanoparticles, structures that are actively ingested by these cells [203,204]. Targeting of liposomes in this manner can be further enhanced if the liposome is coated with ligands for receptors expressed by the cell type of interest [203]. One might conceive of an inactive prodrug that is activated by specific enzymes found within the myelomononuclear phagolysosome [205]. A different strategy could be to use a prodrug compound with minimal electrophilic properties, but which become electrophilic in response to oxidation [206]. This may allow targeting of Nrf2 activation to cells in the oxidative perihaematomal environment. Such central nervous system penetrant cell-type specific Nrf2 activators are in early stages of development and will require preclinical establishment of safety and efficacy. As perihaematomal oedema is only associated with poor outcome at more than three days after ICH, there may be an early window to modulate transcriptional processes associated with oedema and poor outcomes using an Nrf2 activating drug [21,26,27]. Therefore, although side effects have limited the long-term use of non-specific Nrf2 activators for chronic diseases, the required duration of Nrf2 activating therapy may be less after ICH [198]. As such, work towards the conduct of trials of non-specific Nrf2 activators in ICH, whilst targeted drugs are in development, has significant merit.

## 7. Conclusions

ICH is associated with extremely high mortality and morbidity and currently has no effective treatments. After ICH, there is an early phase of transcriptional change and immune cell recruitment. This is associated with perihaematomal oedema, which is associated with worse outcome. Immune cells in the perihaematomal environment have both potentially protective and harmful responses to ICH. Protective responses, include the uptake and detoxification of haematoma breakdown products, and are boosted by activation of the transcription factor Nrf2. Simultaneously, Nrf2 suppresses the expression of proinflammatory factors which may contribute to perihaematomal oedema and poorer outcomes. Studies in rodent ICH models demonstrate improved outcomes with administration of Nrf2 activating drugs. Nrf2 activating drugs in humans have generally favourable safety and efficacy profiles for neurological diseases. To date, no study of pharmacological Nrf2 activation in patients with ICH has been published. Clinical trials of Nrf2 activating drugs, such as DMF and omaveloxolone, for which evidence of safety and efficacy exist for other neurological diseases in humans, seem warranted. Early phase trials for ICH should monitor safety given the older age of patients with ICH, which may be associated with increased susceptibility to potential side effects.

## Figures and Tables

**Table 1:** **Directly Nrf2-regulated genes.** Non-exhaustive list of canonical directly Nrf2-regulated genes, supported by chromatin immunoprecipitation [122,123,156,157,158,159].

Role of Nrf2		Function	Gene Product	Gene Symbols
Transactivation	Haem & iron metabolism	Haem detoxification	Haem oxygenase 1	*Hmox1*
			Biliverdin reductase B	*Blvrb*
		Iron sequestration	Ferritin	*Fth1, Ftl1*
			Haem transporter HRG1	*Slc48a1*
	Glutathione	Glutathione synthesis	xCT cystine antiporter	*Slc7a11*
			Glutamate cysteine ligase	*Gclc, Gclm*
		Glutathione utilization	Glutathione-S-transferase	*Gsta3, Gsta4, Gstm1, Gstm2, Gstm3, Gstm6, Gstm7, Mgst2*
	Thioredoxin	Redox buffering and denitrosylation	Thioredoxin	*Txn1*
			Thioredoxin domain containing 5	*Txndc5*
		Thioredoxin regeneration	Thioredoxin reductase	*Txnrd1*
	Peroxiredoxin	Peroxide reduction	Peroxiredoxins	*Prdx1, prdx6*
		Peroxiredoxin reduction	Sulfiredoxin	*Srxn1*
	NAD(P)H generation and utilization	Production of cofactor for reduction reactions	Pentose phosphate pathway enzymes	*G6pd, Pgd*
		Quinone reduction	NAD(P)H dehydrogenase (quinone 1)	*Nqo1*
Transrepression	Inflammatory signalling	Immune cell activation and recruitment	Interleukin-1β	*Il1b*
			Interleukin-6	*Il6*

**Table 2:** Key in vivo studies of Nrf2 pathway modulation after ICH.

Intervention	Study	Major finding
Global Nrf2 deletion	Wang, et al., 2007 [171]	Larger haematoma volume at 24 h post-ICH and increased neutrophil infiltration
	Zhao, et al., 2015 [172]	Larger haematoma volume at day 7 post-ICH
	Zhao, et al., 2007 [173]	Greater neurological deficit at day 7 post-ICH
Sulforaphane	Zhao, et al., 2015 [172]	Nrf2-dependent reduction in haematoma volume with treatment at day 7 post-ICH
	Yin, et al., 2015 [174]	Reduced neurological deficit from day 1 post-ICH with treatment. Reduced TNF⍺ and NF-𝜅B expression.
Dimethyl fumarate	Zhao, et al., 2015 [175]	Nrf2-dependent reduction in brain water content and Nrf2-dependent improvement in neurological deficit with treatment at day 3 post-ICH. Reduced cytokine and increased haem scavenging protein expression
	Iniaghe, et al., 2015 [176]	Reduced neurological deficit and brain water content from 24 h post-ICH. Reduced myelomononuclear cell recruitment and ICAM1 expression
	Zhao, et al., 2007 [173]	Reduction in day 10 neurological deficit (rats) and Nrf2-dependent day 7 reduction in neurological deficit (mice). Reduced protein oxidation and neutrophil recruitment with treatment.
tert-butyl hydroquinone	Sukumari-Ramesh and Alleyne. 2016 [177]	Reduced oxidative carbonyl production, myelomononuclear cell recruitment, interleukin 1β expression and neurological deficit at 24 h post-ICH with treatment.
RS9 (bardoxolone methyl derivative)	Sugiyama et al., 2018 [178]	Reduced brain water content and haematoma volume from 72 h and improved neurological function from 48h post-ICH with treatment
